# Nkx6.1 decline accompanies mitochondrial DNA reduction but subtle nucleoid size decrease in pancreatic islet β-cells of diabetic Goto Kakizaki rats

**DOI:** 10.1038/s41598-017-15958-6

**Published:** 2017-11-15

**Authors:** Tomáš Špaček, Vojtěch Pavluch, Lukáš Alán, Nikola Capková, Hana Engstová, Andrea Dlasková, Zuzana Berková, František Saudek, Petr Ježek

**Affiliations:** 1 Department of Mitochondrial Physiology, No.75, Institute of Physiology, Academy of Sciences of the Czech Republic, Prague, Czech Republic; 2 Institute of Clinical and Experimental Medicine, Prague, Czech Republic

## Abstract

Hypertrophic pancreatic islets (PI) of Goto Kakizaki (GK) diabetic rats contain a lower number of β-cells *vs*. non-diabetic Wistar rat PI. Remaining β-cells contain reduced mitochondrial (mt) DNA *per* nucleus (copy number), probably due to declining mtDNA replication machinery, decreased mt biogenesis or enhanced mitophagy. We confirmed mtDNA copy number decrease down to <30% in PI of one-year-old GK rats. Studying relations to mt nucleoids sizes, we employed 3D superresolution fluorescent photoactivable localization microscopy (FPALM) with lentivirally transduced Eos conjugate of mt single-stranded-DNA-binding protein (mtSSB) or transcription factor TFAM; or by 3D immunocytochemistry. mtSSB (binding transcription or replication nucleoids) contoured “nucleoids” which were smaller by 25% (less diameters >150 nm) in GK β-cells. Eos-TFAM-visualized nucleoids, composed of 72% localized TFAM, were smaller by 10% (immunochemically by 3%). A theoretical ~70% decrease in cell nucleoid number (spatial density) was not observed, rejecting model of single mtDNA *per* nucleoid. The β-cell maintenance factor Nkx6.1 mRNA and protein were declining with age (>12-fold, 10 months) and decreasing with fasting hyperglycemia in GK rats, probably predetermining the impaired mtDNA replication (copy number decrease), while spatial expansion of mtDNA kept nucleoids with only smaller sizes than those containing much higher mtDNA in non-diabetic β-cells.

## Introduction

The diabetic etiology in Goto Kakizaki (GK) rats stems from multiple aspects of genetic contribution and gestational metabolic impairment inducing an epigenetic programming of the offspring pancreas transmitted over generations^1^, resulting in the reduced β-cell neogenesis and proliferation. Thus, the main etiology of the GK rat diabetes lies in the loss of β-cell differentiation related to chronic exposure to hyperglycaemia/hyperlipidaemia, islet inflammation, oxidative stress, fibrosis and perturbed islet vasculature^1–4^. A striking morphologic feature of GK rat pancreatic islets is represented by large islets with pronounced fibrosis due to connective tissue separating strands of endocrine cells^5–7^. This leads to the spreading of α-cells and δ-cells, forming originally a mantle in non-diabetic rats, within most of the decreased β-cell mass^5–7^.

Recently, β-cell de-differentiation into α-cells has been suggested to participate in human type 2 diabetes etiology^8,9^. A differentiation shift can arise when the expression of certain transcription factors diminishes. A prototype example is Nkx6.1, which controls a gene regulatory network required for establishing and maintaining β-cell identity^10,11^. Nkx6.1 binds to and serves as a repressor for α-cell determinant Arx. In turn, a factor Isl1activates Arx and competes as well with Nkx6.1, consequently establishing balance determining α-cell *vs*. β-cell identity^10,11^. In contrast, the homeodomain transcription factor Hhex (hematopoietically expressed homeobox) is required for δ-cell differentiation^12^. Hhex was previously linked to type 2 diabetes^12^. Hhex deficiency released the paracrine inhibition of β-cell insulin secretion^12^. Interestingly, we have previously found a profound δ-cells hyperplasia increasing with age of GK rats^13^.

Synergy of these pathologic progressions leads to a partial impairment of the glucose-stimulated insulin secretion and to an early progressive development of the peripheral insulin resistance in GK rats^1–4,13^. Since the elevated oxidative phosphorylation in mitochondria is the key component of the β-cell glucose sensor, findings of the reduced amount of mitochondrial DNA (mtDNA)^14,15^ are compatible with the impaired mitochondrial function in GK rats^15^. Using 3D high-resolution 4Pi microscopy, we have found that the mitochondrial network was much more frequently fragmented in β-cells of GK rats, even though mitochondrial volume was preserved^16^. This also indicates a certain kind of stress of yet unknown origin. Also, the disrupted microRNA regulation was found in GK rat β-cells^17^.

Since mtDNA exists in the cell in numerous copies contained in the protein complexes, termed nucleoids^18–27^, we aimed to study how the profound reduction in mtDNA is reflected on the level of nucleoids. mtDNA is compacted in nucleoids by the mitochondrial (mt) transcription factor A (TFAM) in extreme densities. Such densities can be found only in prokaryotic DNA packing^28,29^. Therefore, changes in TFAM have to be investigated. In addition, numerous proteins of the DNA replication and transcription machinery are recruited to nucleoids. Among them the mt single-stranded-DNA-binding protein (mtSSB) is bound on single-stranded mtDNA of the non-coding D-loop region or in replicating and transcribing nucleoids^18,19,30,31^. These particular mtDNA *loci* are unwinded by Twinkle helicase^32,33^. In spite of extensive studies of mtDNA and nucleoids, contradictions between a uniform size^18–20^ and range of nucleoid sizes exist^23–25^; as well as contradictions concerning the number of mtDNA molecules *per* nucleoid^18,19^. Experiments showing the non-existence of mtDNA mixing between nucleotides have indicated one single copy of mtDNA^18,20,34^. However, other findings support an average of six multiple mtDNA copies *per* nucleoid^19^. No particular evidence for a nucleoid division has been observed. However, our preliminary data indicated the possible existence of nucleoid division^24^. Dividing nucleoids must have at least two mtDNA by definition (*i.e*. doubled number of mtDNA molecules *per* dividing nucleoid).

We can theoretically predict how the profound reduction in mtDNA in primary β-cells may be reflected on the level of nucleoids. Assuming the single mtDNA molecule *per* nucleoid, the only obvious variant would exist, lying in the exactly proportional reduction in number of nucleoids. In the model of multiple mtDNA copies *per* nucleoid, also redistribution variants exist. Under the assumption of the same (higher) number of nucleoids within the mitochondrion in two cells despite the mtDNA copy number decrease in one of them, one may imagine that the reduction of mtDNA copies *per* nucleoid might exist in such a cell. A “proportional” variant, reducing the nucleoid number is thus possible for multiple mtDNA copies *per* nucleoid, as well as the “heterogenous” variant of reduced mtDNA molecules only within certain nucleoids. Concerning the nucleoid size, it relies on the definition of a nucleoid^19^. If one considers a nucleoid as the TFAM-contained space, the size can be exactly measured by TFAM-based superresolution microscopy, specifically using 3D imaging^20,21,23,24^. TFAM stabilizes mitochondrial genome^29^ and plays a role of transcriptional function as well^18,19^. TFAM also regulates mt genome copy number^28^. It has also been observed that TFAM overexpression preserves the copy number and respiration as well as ATP synthesis^35,36^. Hence, TFAM-visualized size of nucleoids may change even with constant mtDNA content within nucleoids, or *vice versa*.

We may also consider as well the “mtSSB space” of “active” replicating or transcribing nucleoids encompassing single-stranded DNA-containing regions that should be smaller than the TFAM-confined space^24^. mtSSB ensures replication, repair and maintenance^18,19,31^ of mtDNA, while it binds to the exposed single-stranded mtDNA segments and also promotes mtDNA polymerase^30^ and helicase reactions^32^. Another protein, Twinkle, acting as a mtDNA helicase, unwinds mtDNA in concert with the mtDNA polymerase and mtSSB^32,33^. In the resulting forks, transcription and replication is ensured by specific mitochondrial mtRNA- or mtDNA-polymerases, respectively^18^. Unfortunately, three-strand D-loops exist in each mtDNA molecule, *i.e*. D-loops representing non-coding mtDNA regions and serving as main replication/transcription origins. Hence, a substantial portion of mtSSB molecules is bound in the D-loop locations and represents a high “background” for the active transcribing or replicating nucleoids^24^.

Finally, a nucleoid core is composed of mtDNA^19,24^. Theoretically, under the assumption of the same packing mtDNA density, the observed 75% mtDNA reduction would result in only a 37% decrease of the sphere diameter for the 75% reduced spherical volume. Nevertheless, if the number of mtDNA molecules within a single nucleoid is to be reduced (e.g. from four molecules in the single nucleoid down to a single one), but is simultaneously unpacked, this may also result in a false negative (higher) volume. Thus, spatial immunocytochemistry against DNA will not resolve the problem, unless density of localized points is quantified^24^.

To elucidate the nature of mtDNA pathology changes in Goto Kakizaki PI β-cells, we employed novel 3D superresolution microscopy methods^24^ BiplaneFPALM and direct stochastic optical reconstruction microscopy (dSTORM) and found correlations corresponding to the reduced mtDNA amount in PI β-cells of diabetic GK rats. A theoretical ~70% decrease in nucleoid spatial density was not observed upon a 70% reduced mtDNA copy number, rejecting model of single mtDNA *per* nucleoid. In parallel, we found a profound decline in β-cell-specifying transcription factor Nkx6.1 with age (~12-fold after 10 months) and with increasing fasting hyperglycemia in GK rats. The observed correlation suggests that Nkx6.1 may stimulate mtDNA replication and that the diminished Nkx6.1-mediated maintenance results in decreased mtDNA replication and hence decreased mtDNA copy number in Goto Kakizaki PI β-cells.

## Results

### Diabetic Goto Kakizaki rat pancreatic islets contain much less mitochondrial DNA relatively to Wistar rat controls

**Figure 1:**
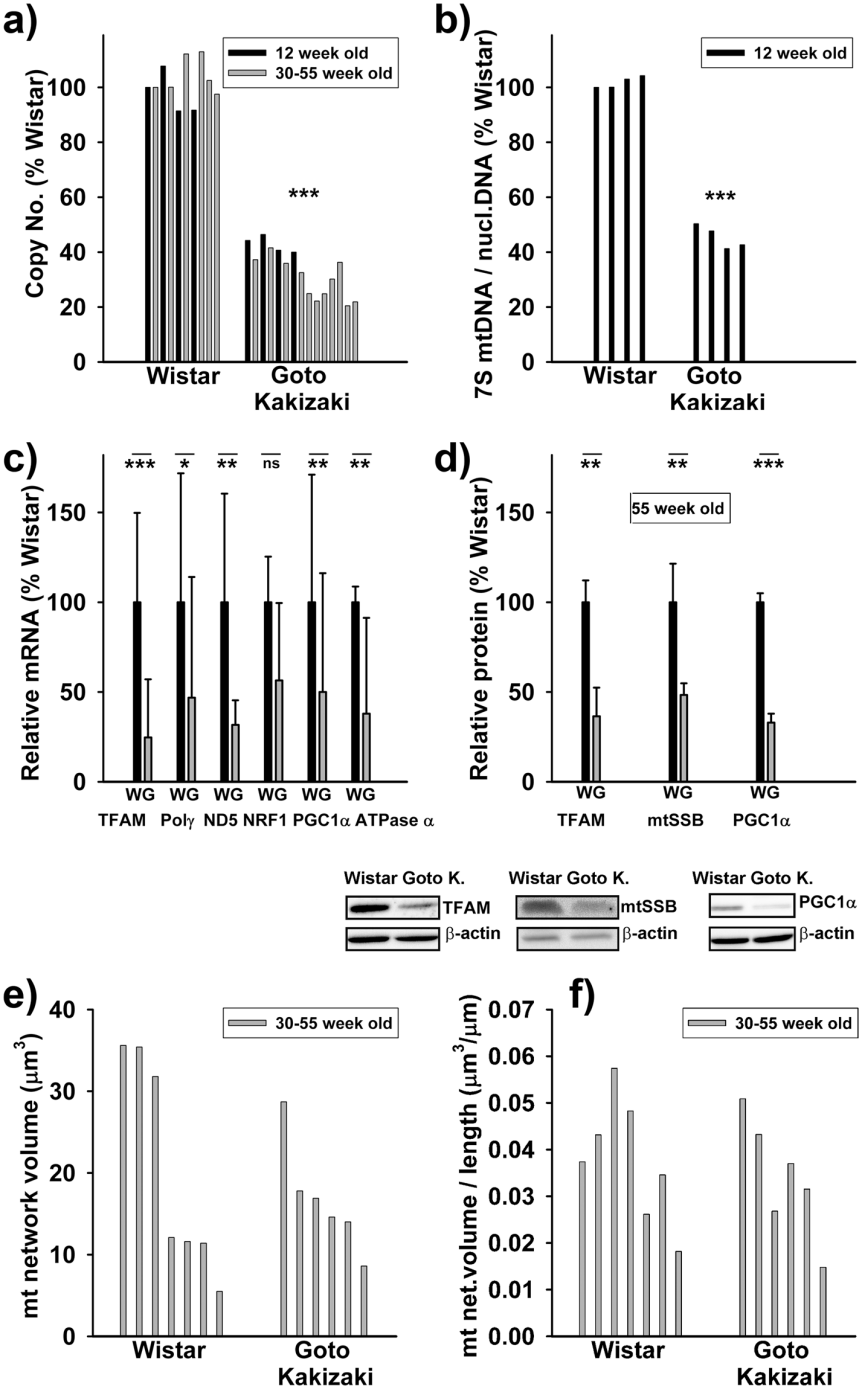
Decreased mtDNA and mtDNA gene expression machinery proteins in PI β-cells of diabetic GotoKakizaki rats. (**a**) **Copy number**; (**b**) 7S DNA/nuclear DNA ratios; (**c**) **amounts** for ascribed nuclear and mtDNA (ND5) **transcripts** for 30–55 week old rats; (**d**) **protein amounts** for selected factors as indicated derived from at least three western blots (typical results illustrated below; here Wistar and GK rat PI samples were always run and are displayed together, while the alternative development for β-actin is displayed below; typical full-length blots and Ponceau Red-stained membranes are included in a Supplementary Information file). (**e**) **Volumes of mitochondrial network** or sum of volumes of all their fragments as derived from 3D 4Pi microscopy images, where each bar represents a single cell (8 cells for each; an average for Wistar rats in μm^3^ was 18 ± 12, median 12, and for GK rats 15 ± 8, median 15); (**f**) **Volume** ***per*** **length of mitochondrial network** of the same data (for Wistar rats an average in μm^2^ was 0.036 ± 0.012, median 0.036, and for GK rats 0.031 ± 0.012, median 0.031). ***P < 0.001; **P < 0.05.

Previously, we reported a 75% decrease of mtDNA (copy number) in primary β-cell cells sorted from the Accutase-digested pancreatic islets (PIs) of diabetic Goto Kakizaki (GK) rats (48 week old), relatively to samples from age-matched non-diabetic Wistar rats^15^. For routine checking of PI samples, we intended to verify, whether such mtDNA decline can be reflected when the entire islets are analyzed. Figure 1a shows the relative copy number decline in PI samples from 12 and 30 to 55 week old GK *vs*. Wistar rats down to 40% and 25–30%, respectively. Also ratios of 7S mtDNA to the nuclear amplicon declined similarly (Fig. 1b).

In parallel, mRNA of transcription factor TFAM and typical mtDNA-encoded transcripts, such as ND5, decreased even more profoundly *vs*. mtDNA down to 25% and 30%, respectively, in 12 week old GK rats (Fig. 1c). A proportional decrease was indicated for mitochondrial polymerase γ (polγ) and ATPase (Fig. 1c), while transcription factor reflecting mitochondrial biogenesis PGC1α declined less (just to half values) in 12 week old GK *vs*. Wistar rats (Fig. 1c). In contrast, NRF1 changed insignificantly. We have also confirmed the similar TFAM, mtSSB and PGC1α decrease in protein levels in the oldest GK rats (Fig. 1d).

In order to relate changes of mtDNA to a “mitochondrial mass”, we employed an analysis of mitochondrial volume as conveniently estimated from our database of 3D high resolution 4Pi images of GK and Wistar rat β-cells^16^. Volume of continuous network in β-cell cells of Wistar rats was insignificantly different as the sum of volumes for fragments of disintegrated mitochondrial network in β-cell cells of GK rats (Fig. 1e). Even the volume *per* a unit length of mitochondrial network/fragment was the same (Fig. 1f). In conclusion, mtDNA was reduced in GK rat β-cells despite the similar mass or volume of mitochondrion.

### Single-stranded-DNA binding protein accumulated in smaller nucleoid compartments in Goto Kakizaki rat β-cells

**Figure 2:**
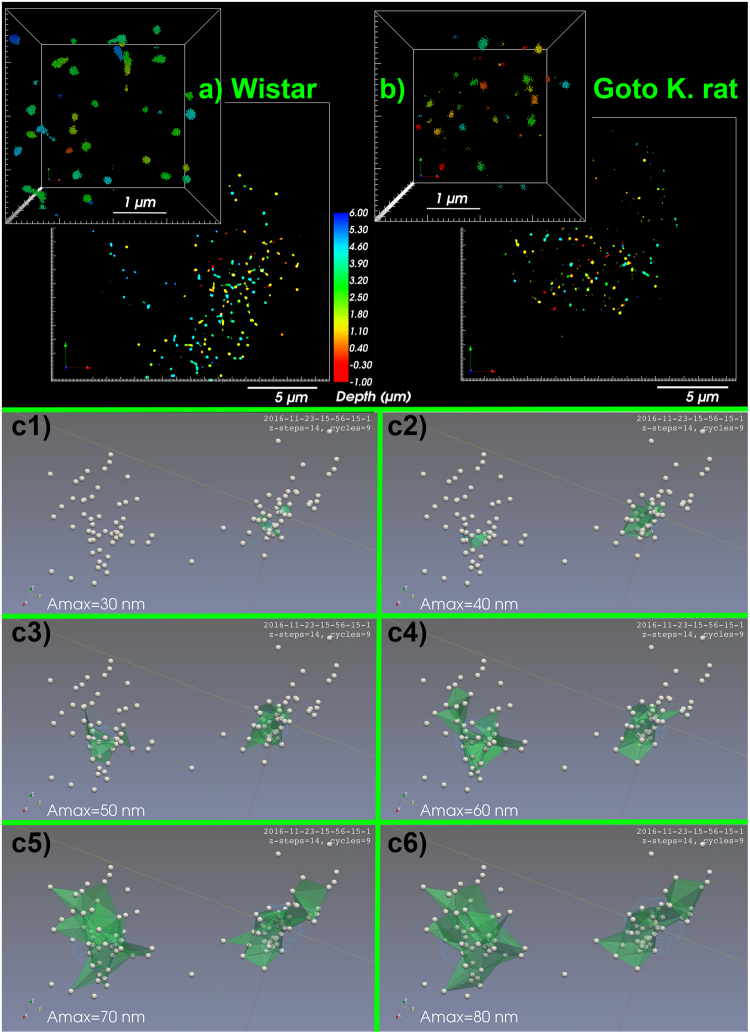
3D BiplaneFPALM images of nucleoids - visualized using mtSSB-Eos (**a**,**b**) 20 × 20 μm xy-projections, depth color-coded in z axis. *Insets*: Detail of 4 × 4 × 4 μm space containing localized points forming nucleoid 3D images. (**c**) **Delaunay tessellation procedure** with increasing base *A*_max_ of tetrahedrons (*green*) in each step **c1**), **c2**)… **c6**).

Employing lentiviral transduction of mtSSB-Eos and 3D superresolution BiplaneFPALM microscopy, we have visualized nucleoids of mtDNA in primary pancreatic β-cells of PI isolated from non-diabetic Wistar control rats and age-matched diabetic GK rats. Among eight PI isolations, raw data indicated each Eos molecule localized within the experiment (Fig. 2a,b). Their grouping into apparent nucleoids^24^ within the equally-sized space regions did not show any significant decline in number (spatial density) of visualized nucleoids (Table 1), despite the profound reduction in mtDNA copy number (Fig. 1a). The nucleoid density within the cell rather increased in β-cells of GK rat PI, with exception of a 10% decrease indicated by dSTORM-assisted 3D TFAM immunocytochemistry (Table 1, *vide infra*).

**Table 1:** Nucleoid density in PI β-cells of Wistar *v*s. diabetic Goto Kakizaki rats.

Nucleoid visualization	Wistar PI β-cells Nucleoid density (μm^−3^)	Goto Kakizaki PI β-cells Nucleoid density (μm^−3^)
mtSSB PALM	0.075 ± 0.040 *n* = 459, *N* = 8	0.123 ± 0.048 *n* = 646, *N* = 6
TFAM PALM	0.067 ± 0.020 *n* = 421, *N* = 4	0.109 ± 0.032** *n* = 1088, *N* = 8
TFAM dSTORM	0.105 ± 0.019 *n* = 740, *N* = 9	0.087 ± 0.014** *n* = 1207, *N* = 9
BrdU dSTORM	0.113 ± 0.044 *n* = 1448, *N* = 13	0.161 ± 0.072 *n* = 820, *N* = 6

^**^P < 0.05; *n* = number of nucleoids; *N* = number of cells.

**Figure 3:**
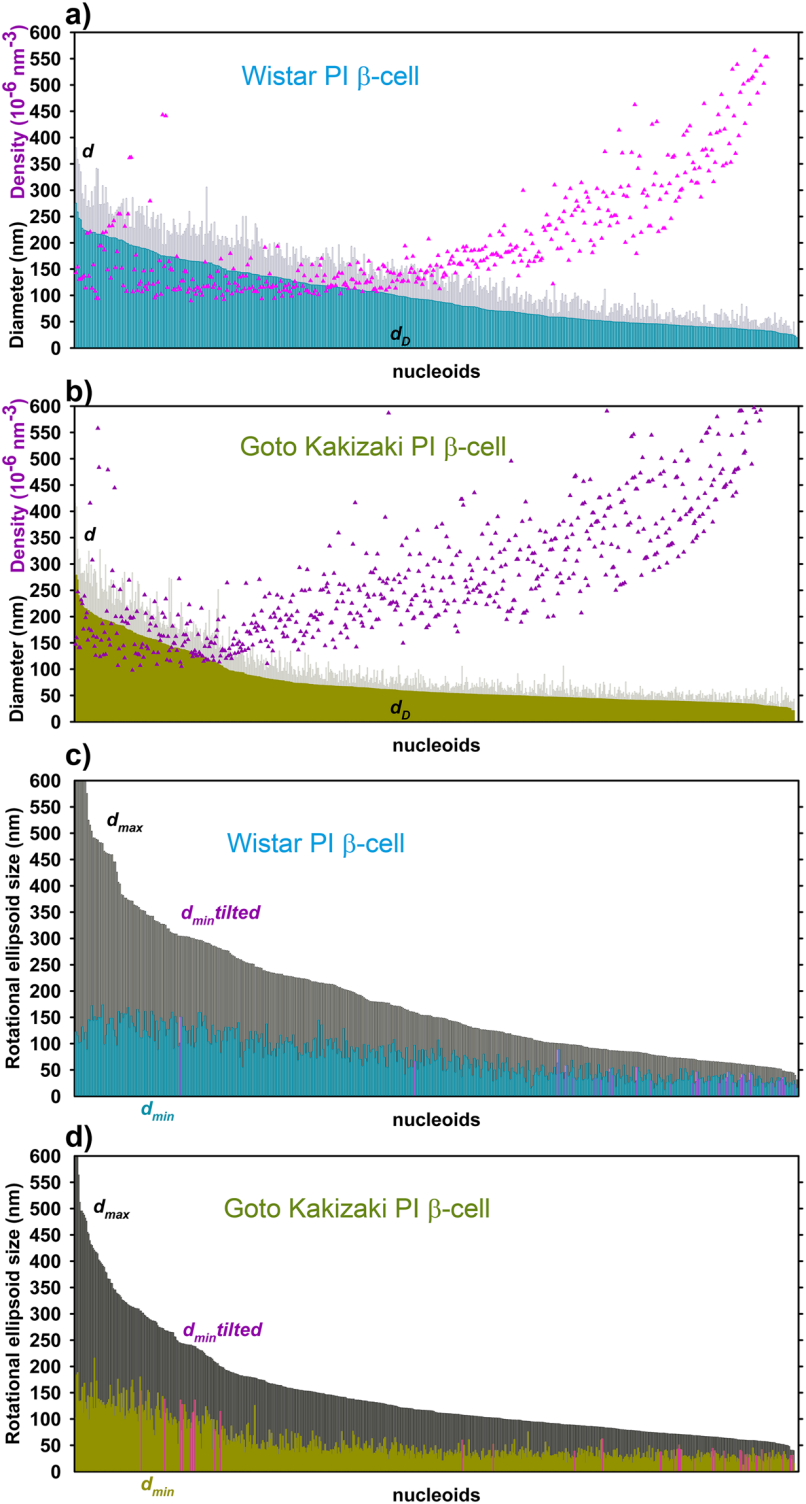
Nucleoids visualized using mtSSB-Eos – (**a**,**b**) spherical models; (**c**,**d**) rotational ellipsoid models - Each nucleoid imaged in several cells is presented in the diagram of nucleoid diameter distributions as a line with size of its diameter *d* (*gray*) or *d*_D_ (color data) for spherical models; or short and long axis diameters *d*_max_ (*black*), *d*_min_ (color data) for ellipsoid models. Wistar PI β-cells (*cyan*, *n* = 458 nucleoid models, *N* = 8 cells, *A*_max_ = 60 nm) were imaged as well as Goto Kakizaki PI β-cells (*dark yellow*, 646 nucleoid models, *N* = 6 cells, *A*_max_ = 60 nm). *Pink data* in **a** (*purple data* in **b**) indicate the localized fluorophore density of each nucleoid model. *Pink data* in (**c,d**) represent ellipsoid models tilted towards x,y-plane (maximum deviation up to 45°).

Therefore, we have performed a thorough data tessellation (grouping) using the Delaunay 3D triangulation, *i.e*. fitting polyhedrons within a group of localized points forming a candidate nucleoid (Fig. 2c). A step *A*_*max*_ (tetrahedron base size) has been properly selected^24^. The resulting nucleoid size distribution plots indicated more frequently smaller mtSSB-Eos-visualized nucleoids in β-cells from PIs of diabetic GK rats, when compared to those of non-diabetic Wistar rats (Fig. 3a,b; *A*_*max*_ = 60 nm). This was valid for both spherical (Fig. 3a,b) as well as ellipsoidal modelling of nucleoids (Fig. 3c,d). Only a few ellipsoids were oriented with their longest axis closer towards the xy-plane (pink data in Fig. 3c,d).

Using the spherical modelling of nucleoid 3D images, *i.e*. when a sphere represents a nucleoid of an equal volume *V* as obtained for the adequate smoothed polyhedron, we obtained an average nucleoid diameter *d* diminished by 27% in PI β-cells from GK rats (*n* = 650) when compared to Wistar rats (*n* = 460) (Table 2). When modelling rotational ellipsoids^24^, we obtained a similar shortening of both long and short axes (Table 2). An alternative is the Delaunay spherical modelling, when diameters *d*_D_ of spheres are set equal to volumes of Delaunay polyhedrons *V*_D_. Such calculations yielded *d*_D_ of 100 ± 57 nm and 76 ± 49 nm for Wistar and GK rat β-cells, respectively. Nevertheless, the resulting 24% diameter *d* decrease represents a 58% decrease in spherical nucleoid volume in GK rat β-cells. Such decrease well matches the mtSSB protein level decline (Fig. 1d). If the corresponding nucleoid volume was filled with mtDNA mass, this should be considered to decrease also by 58%. Expressing averages plus standard deviations or medians (Table 2), however, does not describe the entire statistics. Hence, we analyzed histograms of the data within 10 nm intervals.

**Table 2:** Delaunay model parameters for PI β-cells of Wistar *vs*. diabetic Goto Kakizaki rats – Averages ± s.d. are listed with medians in parentheses. *n* = number of nucleoids and *N* = number of cells are identical as listed in Table 1.

Nucleoid visualization	Wistar PI β-cells Average size in nm (Median)				*Density 10* ^*−6*^ *nm* ^*−3*^	Goto Kakizaki PI β-cells Average size in nm (Median)				*Density 10* ^*−6*^ *nm* ^*−3*^	Goto Kakizaki/Wistar	
	*d*	*d*_D_	*d*_max_	*d*_min_		*d*	*d*_D_	*d*_max_	*d*_min_		For *d*_D_ in %	For *Density* in %
mtSSB PALM	**141 ± **74 (133)	**100 ± **57 (88)	**187 ± **139 (149)	**74 ± **40 (68)	**246 ± **229 (170)	**103 ± **68 (73)	**76 ± **49 (56)	**149 ± **100 (114)	**55 ± **37 (40)	**318 ± **220 (210)	**75%** (63%)	**129%** (124%)
TFAM PALM	**179** ± 108 (165)	**126** ± 73 (113)	**282** ± 209 (221)	**86** ± 48 (79)	**402** ± 229 (271)	**163** ± 82 (158)	**113** ± 56 (111)	**236** ± 158 (212)	**80** ± 39 (78)	**327** ± 414 (195)	**90%** (98%)	**81%** (72%)
TFAM dSTORM	**84** ± 42 (70)	**61** ± 27 (55)	**125** ± 60 (105)	**44** ± 20 (40)	**136** ± 206 (76)	**74** ± 32 (70)	**57** ± 21 (54)	**125** ± 52 (115)	**42** ± 15 (40)	**227** ± 572 (108)	**93%** (98%)	**166%** (142%)

**Figure 4:**
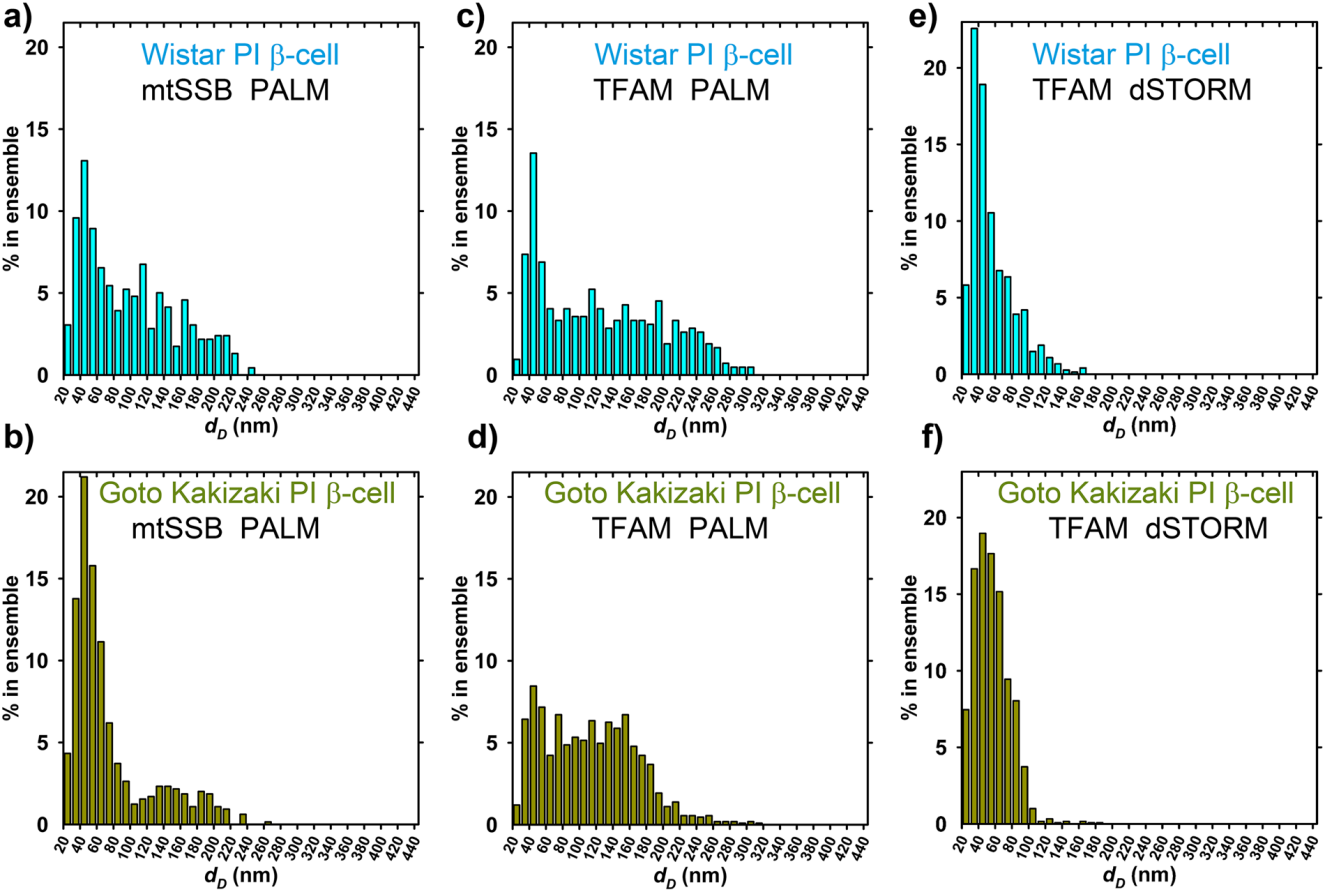
Histograms for diameters *d*_*D*_ of Delaunay spherical models. Wistar PI (*cyan*) and Goto Kakizaki PI β-cells (*dark yellow*) with nucleoid visualized: (**a**,**b**) BiplaneFPALM using mtSSB-Eos (“mtSSB PALM”; data of Fig. 3a.b). (**c**,**d**) BiplaneFPALM using TFAM-Eos (“TFAM PALM”; data of Fig. 5a,b). (**e**,**f**) TFAM dSTORM (data of Fig. 6a,b).

Figure 4a,b shows detailed histograms of Delaunay spherical models for diameters *d*_D_. Histograms for PI β-cells of both Wistar and GK rats contain two maxima at 45 nm and ~135 nm (Fig. 4a,b). Despite the same most frequent values given by these maxima, a fraction of small nucleoids was at least twice as higher for nucleoid models of diabetic GK rat PI β-cells than for Wistar rat PI β-cells (Fig. 4a,b). In contrast, a fraction of 140 to 160 nm nucleoids was diminished more than twice (Fig. 4b). Thus, diabetic GK rat PI β-cells contain less nucleoids with diameter >150 nm and more nucleoids around 50 nm. One may speculate that these largest nucleoids are actually twins representing dividing nucleoids^24^. One may also consider these mtSSB-containing regions as the nucleoid cores^19^. Since mtSSB is considered to reflect mtDNA replication (or transcription), we may conclude that the portion of replicating mtDNA is much less abundant in diabetic Goto Kakizaki rat PI β-cells.

However, the density of localized Eos-mtSSB molecules within a single nucleoid fit into a wide range independently of nucleoid size. This range was slightly wider and on average 1.25-fold higher for GK PI rat PI β-cells (Fig. 3a,b; Table 2). Thus within an ensemble,”pointilistic” nucleoid images were composed of both dense and more diffuse points for PALM imaging, whereas the lower and narrow-range point density was characteristic for dSTORM imaging of larger nucleoids (*vide infra*). In conclusion, the resolved mtEos-mtSSB-contoured “nucleoids” were by 25% smaller but appear to be 1.25 times denser in β-cells of PI isolated from GK *vs*. Wistar rats. Since the density of blinking molecules might increase partially due to a repeatable occurrence of a molecule slightly shifted meanwhile during the data acquisition, one should consider density calculations with a caution.

### Slight decrease in TFAM-visualized nucleoid volume but a drop in localized point density in diabetic PI β-cells

**Figure 5:**
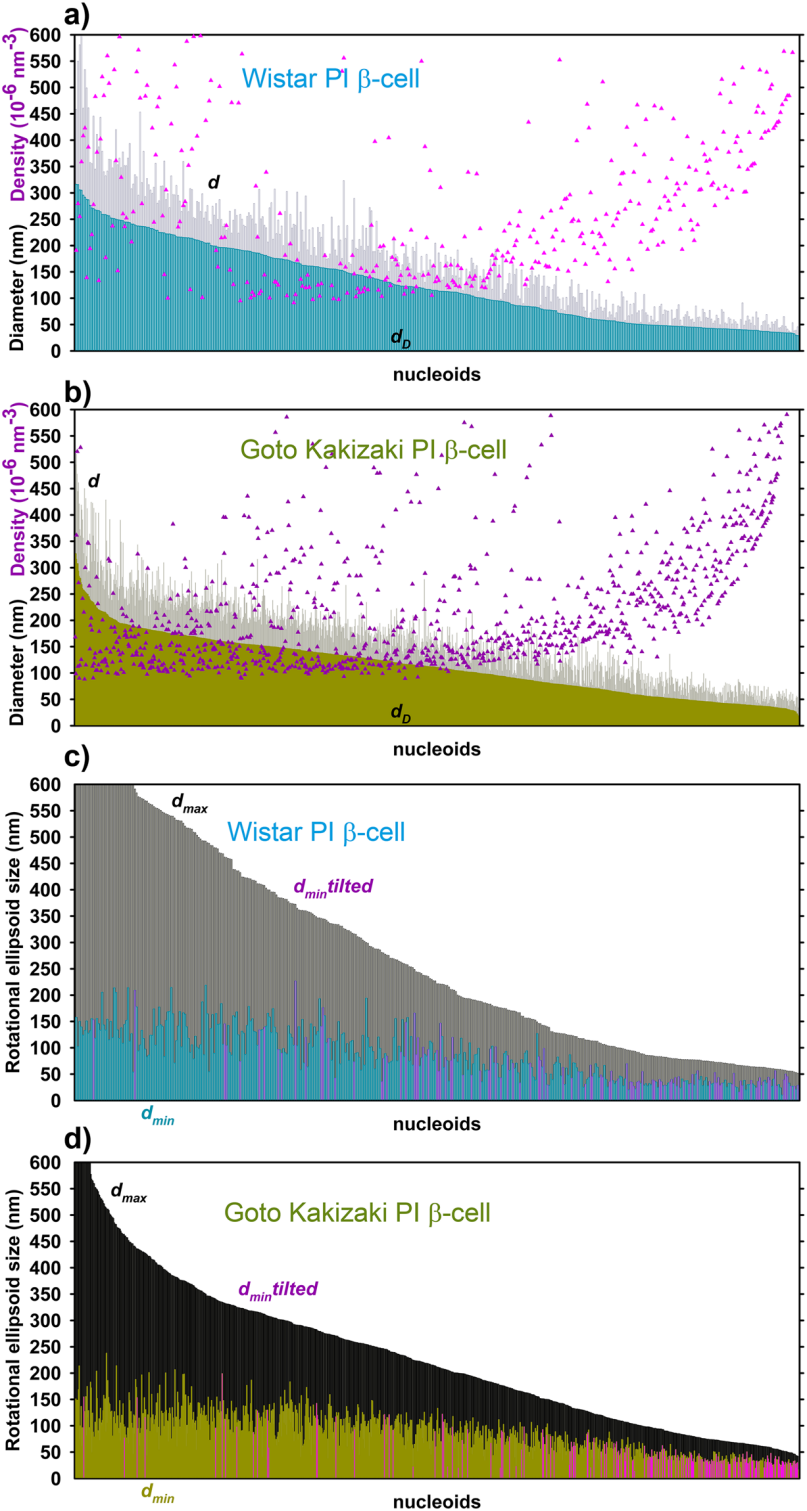
Nucleoids visualized using TFAM-Eos. –(**a**,**b**) **Delaunay spherical models**, (**c**,**d**) **rotational ellipsoid models**. *A*_max_ = 60 nm. *n* was 421 in (**a**) and 1088 in (**b**) within 4 and 8 cells, respectively. Color coding see Fig. 3 Legend.

Much less differences were found for nucleoid size distribution, when visualized by Eos-TFAM, lentivirally-transfected into primary β-cells of PIs isolated from non-diabetic Wistar control rats and diabetic GK rats (Fig. 5a–d; Table 2). TFAM-contained space within the nucleoids was bigger than the mtSSB-contained space, as already recognized for HepG2 cells^24^. Medians for *d*_D_ were equal, while averages of *d*_D_ were only by 10% smaller in PI β-cells of GK rats *vs*. those for Wistar rats (Table 2). Detailed histograms of Delaunay spherical models for diameters *d*_D_ again indicated two maxima at 45 nm and ~135 nm (Fig. 4c,d). Here, the maximum fraction of small ~45 nm nucleoids had nearly equal proportions in both Wistar rat PI β-cells (Fig. 4c,d). A fraction of ~140 to 160 nm nucleoids was even slightly more frequent in diabetic Goto Kakizaki rat PI β-cells. This may represent an expansion of TFAM-acquired space. Wistar rat PI β-cells, however, had more nucleoids (clusters) of sizes exceeding 200 nm. In contrast, the average density of localized TFAM molecules within a single nucleoid diminished by 19% (by 28% for medians) in PI β-cells of GK rats, which may reflect up to ~30% loss of total TFAM. Note that mRNA or protein semiquantification of TFAM yielded even larger decrease (Fig. 1c,d).

**Figure 6:**
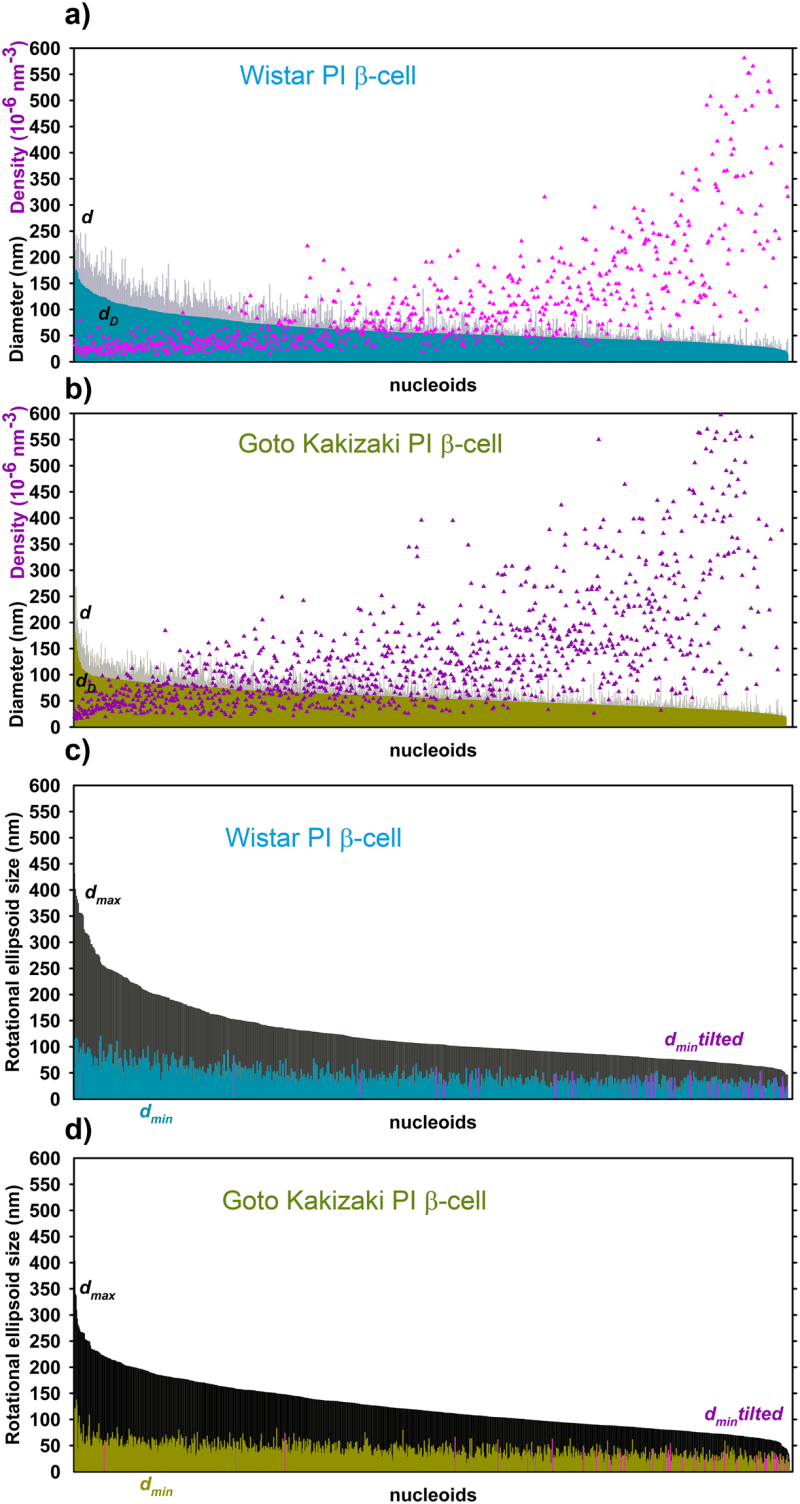
Nucleoids visualized dSTORM with primary antiTFAM antibodies. (Alexa Fluoro 647 conjugated) (**a**,**b**) **Delaunay spherical models**, (**c**,**d**) **rotational ellipsoid models**. *A*_max_ = 80 nm. *n* was 740 in (**a**) and 1207 in (**b**); *N* = 9. Color coding see Fig. 3 Legend.

Overexpression of Eos-conjugated proteins employed in the above experiments also tests the vitality of isolated PIs, indicating functional protein expression machinery. To make a snapshot of native TFAM protein present, we have alternatively employed *anti*-TFAM antibodies in 3D dSTORM-assisted immunocytochemistry with Alexa Fluor 647 conjugated secondary antibodies (Fig. 6a–d; Table 2). The resulting histograms for diameters *d*_D_ of Delaunay spherical models, however, exhibited again a single most frequent diameter *d*_D_ around 45 nm in both Wistar and Goto Kakizaki PI rat β-cells (Fig. 4e,f). The only differences in histograms were apparent as less population of nucleoids >100 nm in Goto Kakizaki rat PI β-cells. However, within ensembles of nucleoids for GK samples, dSTORM yielded by 10% lower number of nucleoids *per* cell volume (Table 1), despite only a subtle decline in sizes – diameters *d*_D_ or any parameters of ellipsoid models (Fig. 6a–d; Table 2).

### The lack of biogenic transcription factors may reduce/impair mtDNA replication machinery in GK rat PI β-cells

**Figure 7:**
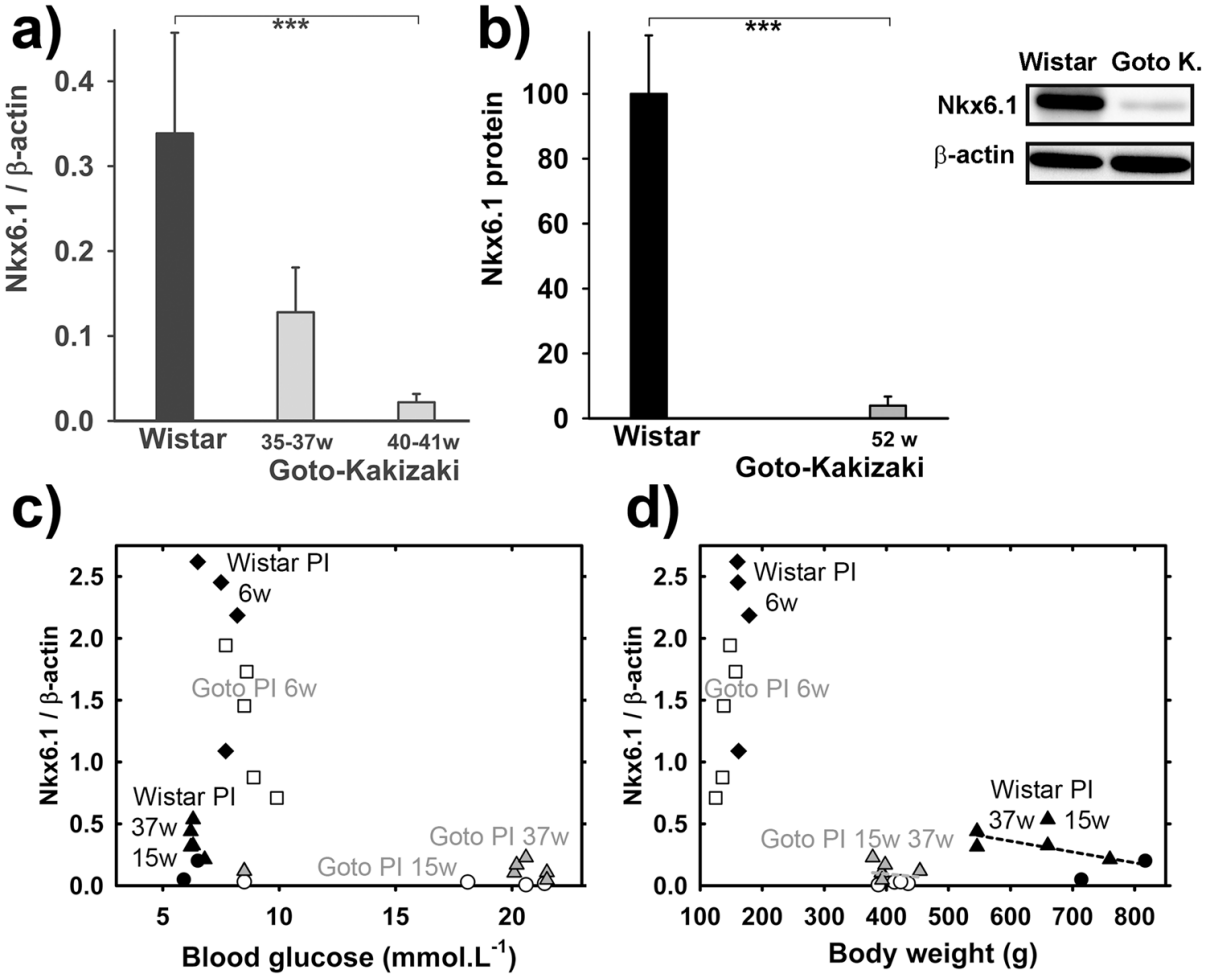
Assays of β-cell–specifying transcription factor Nkx6.1. (**a**) **Relative Nkx6.1 to** β**-actin ratio** in PI of Wistar (*black*) and GK rats (*gray*). (**c,d**) Nkx6.1/β-actin transcript ratio in PI *vs*. blood glucose (**b**) or body weight (**c**) in Wistar (*black*) and GK rats (*gray* and *white*) at age 6 (white squares; black diamonds); 15 (circles) and 37 weeks (triangles). ***P < 0.001. (**b**) **Protein amounts** for Nkx6.1 as derived from five Western blots. Wistar and GK rat PI samples were always run and are displayed together (as illustrated), while the alternative development for β-actin is displayed below. Typical full-length blots and Ponceau Red-stained membranes are included in a Supplementary Information file).

Previously, we reported on δ-cell hyperplasia developed after six weeks of age in PI of diabetic GK rats. This finding with the well established decrease of β-cells in PI of GK rats with age (and their peripheral insulin resistance developed already prior to the six weeks of age) suggest that the impaired or deregulated biogenesis of islet cells and resulting disharmony in their paracrine relationships contribute to the diabetic phenotype. To further elucidate it, we assayed for a β-cell-specifying transcription factor^12,13^ Nkx6.1. In PI of adult GK rats, and of less extent in 6-weeks old, we found a profound decrease of Nkx6.1 transcript. Thus, RT PCR assessment (relatively to β-actin) in isolated PI at weeks 35, 37, or 39–41 has shown a ~12-fold decrease of Nkx6.1 mRNA (Fig. 7a) in GK rats as compared to Wistar controls. Similar decrease was found for Nkx6.1 protein (Fig. 7b). Young, six week old GK rats exhibited still high Nkx6.1 mRNA levels but 60–75% of those in Wistar rats (Fig. 7c,d). In both, non-diabetic and diabetic rats, Nkx6.1 decreased with age but more profoundly in diabetic GK rats, for which turned out to be a good marker of hyperglycemia (Fig. 7c). For both rat strains reciprocal correlation was found between Nkx6.1 mRNA levels and body weight (Fig. 7d).

We have also confirmed a decrease in protein levels of Nkx6.1 with age, while a more intensive decline was found for old GK rats (Fig. 7b).

## Discussion

Organization of mtDNA within the nucleoids is still a matter of debate^18,19^, as well as detailed changes during the typically physiological situations, including mtDNA transcription and mtDNA replication. It is not known, whether, when and how nucleoids divide, despite the first snapshots of such possible events have been reported^24^. Variations of TFAM-contoured nucleoid size have been reported under numerous pathological conditions as well as decline of mtDNA copy number^37,38^. However, when conventional confocal microscopy was used, one could not distinguish single nucleoids from nucleoid clusters. We do not know, whether cells with lower mtDNA copy number should contain less nucleoids or nucleoids with a smaller size or a combination of both.

In this work we have elucidated some of these aspects in the case of a highly reduced (by 75%) mtDNA copy number, as found in PI β-cells of one year old Goto Kakizaki rats^15^. The apparent mitochondrial volume, however, remained the same. Either nucleoid diameter reduction by ~37% or nucleoid number by 75% or combination of both was expected. Surprisingly, the prediction of lower nucleoid number turned out to be invalid. We have even obtained an increased nucleoid spatial density with some employed markers; alternatively a 10% decrease was observed by 3D TFAM immunocytochemistry (Table 1). In contrast, the prediction of 37% reduction of nucleoid size at 75% mass losses turned out to be reflected only when employing mtSSB protein for visualizing nucleoid cores which might be active in mtDNA replication and/or transcription. In principle, such visualization indicates the extent of mtDNA cores containing single-stranded DNA regions, which are much smaller than the TFAM-containing regions^24^. Using spherical modelling, volumes of these regions decreased on average by ~25%, corresponding to a ~58% mass (mtSSB bound to ss mtDNA) reduction (cf. Figure 1a,b). This reduction was also matched by western blots, indicating more than 50% mtSSB decline. Diabetic GK rat PI β-cells contained less nucleoids with diameter >150 nm but more small nucleoids (around 50 nm). Thus the detailed diameter histograms indicated shift from 140 nm to 160 nm regions down to a <100 nm region for GK rat PI β-cells when compared to Wistar controls. However in these smaller regions, more dense Eos-mtSSB molecules were bound. A partial contribution of a drift of blinking molecules (though existing in both types of samples) might artificially increase such density. We may conclude that the regions with any “active” mtDNA (transcribing or replicating, *i.e*. requiring Eos-mtSSB) within the nucleoids are diminished in diabetic GK PI β-cells.

In the case of TFAM-Eos, GK PI β-cells contained less nucleoids in the range over 200 nm that might represent rather clusters of several nucleoids or even dividing nucleoids^24^. Moreover, GK rat PI β-cells had reduced density of localized points (blinking Eos molecules) within model nucleoids with only slightly diminished nucleoid diameters. This might indicate expansion of mtDNA with similar or lower stoichiometric TFAM amount.

The model of single mtDNA molecule within a single nucleoid is rejected by our results. In any of multiple imaging ways, the nucleoid number (exactly spatial density, see Table 1) rather increased despite the diminished mtDNA copy number. Our preliminary data counting D-loops visualized by a poly-fluorophore DNA probe against the 7S DNA indicate the existence of several nucleoid fractions, nucleoids having 2, 3, 4, 5 and even in ~1% fraction 6 D-loops within a single nucleoid (Capková N, unpublished data), beside the prevailing fraction with single D loop. When considering the single mtDNA *per* nucleoid, those nucleoids with multiple D-loops should represent nucleoids under mtDNA replication. However, the maintained nucleoid number at drastic copy number reduction cannot be explained.

We have also pointed out to the link between the decreased mtDNA in diabetic β-cells and even more declining β-cell identifying and maintenance transcription factor Nkx6.1 (down to ~8%). Although Nkx6.1 transcript decreased with age in both non-diabetic Wistar and diabetic Goto Kakizaki rats, the decrease was much stronger in PI of diabetic rats (the oldest tested exhibited Nkx6.1 protein down to ~4%). Since Nkx6.1 is controlling a gene regulatory network required for establishing and maintaining β-cell mass within PIs^10,11^, its negative effect on factors and proteins required for mitochondrial biogenesis is expected. More drastic Nkx6.1 decline above that occurring naturally with aging must impair β-cell function. Although mitochondrial biogenesis factors such as PGC1α decreased in less extent, synergy of these housekeeping and biogenetic impairments must affect the resulting mtDNA replication and content. This corresponds also to lowered transcript of mitochondrial polγ. Previously, also a diabetic phenotype has been found in aged mice with ablated β_2_-adrenergic receptor accompanied by the reduced expression of peroxisome proliferator-activated receptor (PPARγ), pancreatic duodenal home homeobox-1 (PDX-1) transcription factor, and glucose transporter (GLUT2)^39^.

Finally, we would like to point out that the mitochondrion exists in the cell (the single network of mitochondrial reticulum in intact cells) and that the term mitochondria represents, in fact, the existing fragments of the network when fission prevails over fusion^40^, such as found in diabetic Goto Kakizaki rats^16^. The isolated mitochondria are then produced from the artificially cut network. Hence the term mitochondrial mass must refer to the mass of all fragments of the network or to the whole continuous network and can be approximated by the mitochondrial volume as we derived from our 3D 4Pi high resolution microscopic images. We prefer this approximation of “mitochondrial amount” to measurements of aconitase activity or marker proteins which have some limitations^41^.

In conclusion, we hypothesize that the diminished Nkx6.1-mediated maintenance results in decreased mtDNA replication and hence decreased mtDNA copy number in diabetic Goto Kakizaki PI β-cells. However, mtDNA is more spread within the single nucleoid, consequently maintaining their size at least in a fraction of a nucleoid ensemble.

## Methods

### Vector Constructs for nucleoid visualization

The pLenti6.3/V5-DEST vector (Life Technologies, currently Thermo Fisher Scientific, Waltham, MA) has been adopted for conjugation of any ORF with the dimerizing Eos. A single Eos sequence was amplified from pcDNA3-Flag1-td-EosFP vector (MoBiTec Rastatt, Germany) and subcloned behind the attR2 site into the pLenti6.3/V5-DEST using *Xho*I restriction site giving rise to pLenti6.3-C-EOS-V5-DEST enabling c-terminal fusion with dimeric Eos sequence after LR-recombination reaction, since ptd-EostFP encodes dimeric Eos protein^42^ due to the T/R substitution of wt Eos. Expression thus causes dimerization of the fusion protein with its other dimeric counterpart. The resulting vector enables C-terminal fusion of protein of interest with respective fluorescent protein. Accordingly, the entry clones (ORFs in pENTR221 vector, Life Technologies/Thermo Fisher) of TFAM (ID IOH42148), and mtSSB (ID IOH63190), were subcloned into our pLenti6.3-C-EOS-V5-DEST vectors using LR-recombination reaction.

### Lentiviral particle production

The pLenti6.3-C-EOS-V5-DEST-based constructs have been multiplied, purified and used for lentiviral particle production according to the manufacturer instructions. Thus the lentiviral stock (containing the packaged pLenti expression construct) was produced by co-transfecting the optimized packaging plasmid mix (packaging plasmids pLP1, pLP2, and pLP/VSVG, which supplies the helper functions as well as structural and replication proteins required to produce the lentiviruses) and our expression construct into the 293LTV cell line, a derivative of the 293F Cell Line. The 293 LTV cell line is stably and constitutively expressing the SV40 large T antigen facilitating an optimal lentivirus production. Lipofectamine 2000 (Thermo Fisher) was used as the transfection reagent. The lentiviral stock was filtered and concentrated by PEG-it Virus Precipitation Solution or using its separation by ultracentrifugation.

### Animals and islets isolation

Wistar rats and Goto-Kakizaki rats (GK/MolTac, Taconic Farms, Inc., Hudson, NY, USA) were bred in accordance with the European Guidelines on Laboratory Animal Care and according to the Institute of Clinical and Experimental Medicine licensing committee (Committee for Animal Welfare, IKEM) and the Czech Ministry of Health approval No. 12782/2015-OVZ-30.0-12.3.15. PI isolation was performed according to a standard protocol^13,15^ in parallel from both groups one by one in the course of observation on the indicated weeks.

### qRT-PCR

Primers were designed with the help of NCBI Design Primer tool - for Nkx6.1: *5′-GGA GAG TCA GGT CAA GGT CTG-3′* (forward); *5′-TCT CGT CGT CAG AGT TCGGG-3′* (reverse), for TFAM: *5′-GCA TGA TAA CAA GCC CCT GGA-3′* (forward); *5′-CCA GTG TTC TTA GCA CGCCC-3*′ (reverse), for POLG: *5′-ATG CGA ATG GTC CAG CGA TTT-3′* (forward); *5′-AAC AGT TCC CGA GGC TCCTT-3′* (reverse), for ND5: *5′-AAC TCC CGT CTC TGC CCTAC-3′* (forward); *5′-GGC CTA GTT GGC TGG ATGTT-3′* (reverse), for NRF1: *5′-GCT AAT GGC CCA GAT GGA GTT-3′* (forward); *5′-CGT AAG CTC TGC CTG GTTGT-3′* (reverse), for PGC1: *5′-TGG AGT GAC ATA GAG TGT GCTG-3′* (forward); *5′-CGC GGG CTC ATT GTT GTACT-3′* (reverse), for ATPase-α: *5′-TCC AAG CAG GCT GTT GCT TAC-3′* (forward); *5′-TGT AGG CGG ACA CAT CACCA-3′* and for rat β-actin: *5′-CCA CAC CCG CCA CCA GTTCG-3′* (forward); *5′-GGC CCG GGG AGC ATC GTC-3′*(reverse). The PCR reaction was performed in LightCycler 480 (Roche) utilizing Maxima SYBR Green qPCR Master Mix (Pierce Biotechnology, Meridian Road Rockford, IL). The absolute mRNA amounts were calculated from crossing points of each run.

### mtDNA copy number estimation

The mtDNA from PIs or primary β-cells was isolated by phenolchloroform extraction. SYBR Green qPCR amplification used primers annealing on the UCP2 nuclear gene (intron 2 and exon 3) and the ND5 mitochondrial gene (bp 11092 to 11191 according to Genebank sequences from The National Center for Biotechnology Information, USA). Alternatively, 7S mtDNA sequence bp 15412 to bp 16309 bp was used to include mtDNA with possible deletions. The ratio between ND5 amplicon (7S mtDNA) and half of nuclear amplicon amounts was taken as the mtDNA copy number *per* cell.

### Western blotting

Cells (Islets) were lysed in RIPA buffer (150 mM NaCl, 50 mM Tris, 1 mM EGTA, 1 mM EDTA, 0,1% SDS, 0,1% Deoxycholate, 1% Triton X-100, 1 mM PMSF, pH 7,6) under constant agitation at 4 °C. for 30 min. The lysate was centrifuged at 16 000 g for 20 minutes to remove debris and the supernatant was used for protein quantification by bicinchoninic acid assay. Proteins separated on SDS-polyacrylamide gels were transferred by a wet electroblot onto PVDF membranes, treated subsequently with the relevant primary antibody followed by the secondary (horseradish peroxidase-conjugated) antibody. ECL detection was accomplished with a Luminata Forte Western HRP Substrate (Millipore Corporation, Billerica, MA 01821), with antibodies against Nkx6.1 (Cell Signalling Technology, Boston, MA, 54551), PGC1 (ThermoFisher, PA5–38021), TFAM (Abcam, ab119684). ECL light intensity was quantified by densitometry with FIJI.

### Biplane FPALM microscopy

Islets were cultured on #1.5H coverslips coated with poly-d-lysine and transfected with the corresponding lentiviral particles and fixed with 4% paraformaldehyde. Imaging was performed on our Biplane FPALM instrument, a prototype of Vutara (formerly Vutara Inc., Salt Lake City, UT; now Bruker Nanosurface, Middleton, WI), equipped with lasers (Coherent, Santa Clara, CA) emitting at 405 nm (Obis 405), 488 nm (Sapphire 488–200), 561 nm (Sapphire 561–200) and 641 nm (Cube 640–100). Light from lasers is collimated and sent through an acousto-optical tunable filter (AOTF R64040, Gooch & Housego, Ilminster, UK) to a multimode fiber. Emerging light from the fiber is reflected by a multiband dichroic beamsplitter (Di01-R405/488/561/635, Semrock, Rochester, NY) onto the back aperture of a 60×/1.2NA water immersion objective (UPlan-SApo 60×/1.2w, Olympus, Center Valley, PA) mounted on a piezo objective scanner (P-725 PIFOC, Physik Instrumente, Karlsruhe, Germany). The collected fluorescence from the sample is filtered using a bandpass emission filter (FF01-446/523/600/677-25, Semrock, Rochester, NY) and split in front of an EM-CCD camera (Evolve 512, Photometrics, Tuscon, AZ) to create two images of different focal planes^43,44^. Recorded raw images were localized and then analyzed by the Vutara SRX software package using algorithms similar to the ones previously described^43^. Localized data were subjected to accuracy based filtering and a drift correction^44^.

For GK PI only the cells stained for β-cell-selective markers (see below) were imaged. Eos fluorophore of conjugate marker proteins was activated with the 405 nm laser line and Eos red fluorescence was read out as excited with the 561 nm laser line at ~5 kW/cm^2^. Two raw images (512 × 256 pixels, 16 bit) were taken at rate 33 frames *per* second. To ensure an even distribution of detected molecules over the entire axial range, the objective position was stepped at 500 nm intervals over a range of about 3 μm for the selected cell. The axial scanning procedure was therefore repeated several times with only a fraction of the molecules activated during each cycle. This ensures nearly constant particle yields over the complete axial range. Measurement took place over the course of several minutes and resulted from ten up to ~1500 of localized fluorophores *per* nucleoid. The average photon count from a single signal detected and localized was 400. The average number of events *per* frame was 6.2.

### 3D immunocytochemistry by dSTORM

Cells were fixed by 4% paraformaldehyde for 10 min and washed twice in phosphate buffered saline (PBS). Further steps were done in “washing PBS” containing 0.05% Triton ×100, 0.05% Tween 20, and 0.1 M glycine. The fixed cells were blocked by 5% donkey serum (Jackson ImmunoResearch, West Grove, PA) for one hour and then the selected primary antibodies were applied, rabbit *anti*-insulin and mouse *anti*-TFAM antibodies (Abcam/Thermo Fisher); alternatively, after the bromo-deoxyuridine (BrdU) pretreatment prior to fixation, mouse *anti*-BrdU antibodies (Progen Biotechnik, Heidelberg, Germany) were applied. Coverslips were then washed three times in “washing PBS” and incubated with donkey *anti*-rabbit and *anti*-mouse IgG secondary antibody conjugated with Alexa Fluor 488 and 647, respectively (Life Technologies/Thermo Fisher). Finally, samples were washed three times in PBS and mounted in the “dSTORM buffer” (10% glucose, 50 mM β-mercaptoethanol, 169 units of glucose oxidase, 1.4 units of catalase all in 10 mM NaCl, 50 mM Tris-HCl (pH 8.0). Only Alexa Fluor 488 positive cells were imaged. Imaging was performed using the BiplaneFPALM instrument at 641 nm, 3.5 kW/cm^2^. The average photon count from a single signal detected and localized was ~1400 for *anti*-TFAM; ~2300 for *anti*-BrdU. The typical number of events *per* frame was around 1.

### Single nucleoid determination from 3D data

3D image reconstruction was done as described elsewhere^24^. Paraview software (www.paraview.org) was used including Python programming language modules for tetrahedron parameter functions, thus ensuring filtering or drawing of bounding ellipsoid. Routinely in each image, only nucleoids constructed from more than 10 localized Eos or Alexa Fluor 647 molecules were considered. Sets of localized particles were segmented into individual nucleoids by Delaunay triangulation algorithm^45^. The resulting convex hulls were filled with the corresponding tetrahedrons (pyramids) with the base of size *A*_max_ (in nm). Tetrahedrons with all edges shorter than the threshold value *A*_max_ represented rough nucleoid models, where an individual nucleoid is given by polyhedrons given by a set of connected tetrahedrons (Fig. 2c). For *spherical modelling* the resulting polyhedrons were approximated by spheres of the equal volume as either *i*) the resulting polyhedron for each nucleoid (volume *V*_D_ yielding the corresponding diameter *d*_D_); or *ii*) smoothed polyhedron with tetrahedrons added to get convex shapes (volume *V* yielding the corresponding diameter *d*). *Ellipsoid modelling* was also performed as further refinement based upon the Principal component analysis. Rotational ellipsoids were illustrated with a volume equal to *V*_D_ (detailed description see^24^), yielding the diameter of the longest and the short ellipsoid axis *d*_max_ and *d*_min_. We have also taken account of ellipsoid orientation, given by unit space vectors oriented as the the longest ellipsoid axis. Their axial projections are denoted as *a*_x_, *a*_y_, *a*_z_. Defining θ as the angle between the longest ellipsoid axis and z axis, we have sorted out the nucleoids oriented with the longest axis tilted more towards the xy plane as those with θ < 45° Since, it is valid that θ = *arctg* [(*a*_x_^2^ + *a*_y_^2^)^1/2^/*a*_z_], the ratio |*A*_*r*_ **/** *a*_*z*_| has to be higher^24^ than 1 for θ < 45°.

### 3D 4Pi microscopy and image analysis

3D images were obtained and analyzed essentially as described elsewhere^16^. Briefly, custom measurements at the Jackson laboratory, Bar Harbor, MN with a Leica TCS 4PI microscope have been performed with GK and control Wistar PI samples lentivirally transduced with mtRoGFP. Images were visualized in 3D projections created with Amira 5.3 (FEI, originally Visage Imaging, Berlin, Germany) in an iso-surface mode. The intensity threshold IT for surface rendering was set to 30–35 to obtain similar tubule diameters as measured from the original data^16^. Volumes of the continuous or fragmented mitochondrial network were derived with the aid of the Autoskeleton function.

### Data availability statement

The datasets generated during and/or analysed during the current study are available from the corresponding author on reasonable request.

## Electronic supplementary material

Dataset 1
