# Supplementary material for: Nkx6.1 decline accompanies mitochondrial DNA reduction but subtle nucleoid size decrease in pancreatic islet β-cells of diabetic Goto Kakizaki rats
Source: Sci Rep. 2017 Nov 15;7:15674. doi: 10.1038/s41598-017-15958-6 (PMC5688109; doi:10.1038/s41598-017-15958-6)
Supplement: Supplementary file 1 — Dataset 1 [file 41598_2017_15958_MOESM1_ESM.doc]

**SUPPLEMENTARY INFORMATION**

**Nkx6.1 decline accompanies mitochondrial DNA reduction but subtle nucleoid size decrease in pancreatic islet β-cells of diabetic Goto Kakizaki rats**

Tomáš Špaček**^1^**, Vojtěch Pavluch**^1^**, Lukáš Alán**^1^**, Nikola Capková**^1^**, Hana Engstová**^1^**, Andrea Dlasková**^1^**, Zuzana Berková**^2^**, František Saudek**^2^**, and Petr Ježek**^1*^**

**^1^***Department of Mitochondrial Physiology, No.75, Institute of Physiology, Academy of Sciences of the Czech Republic, Prague, Czech Republic*

**^2^***Institute of Clinical and Experimental Medicine, Prague, Czech Republic*

***Corresponding author**

**Supplemental Figure S1 Expression of TFAM**
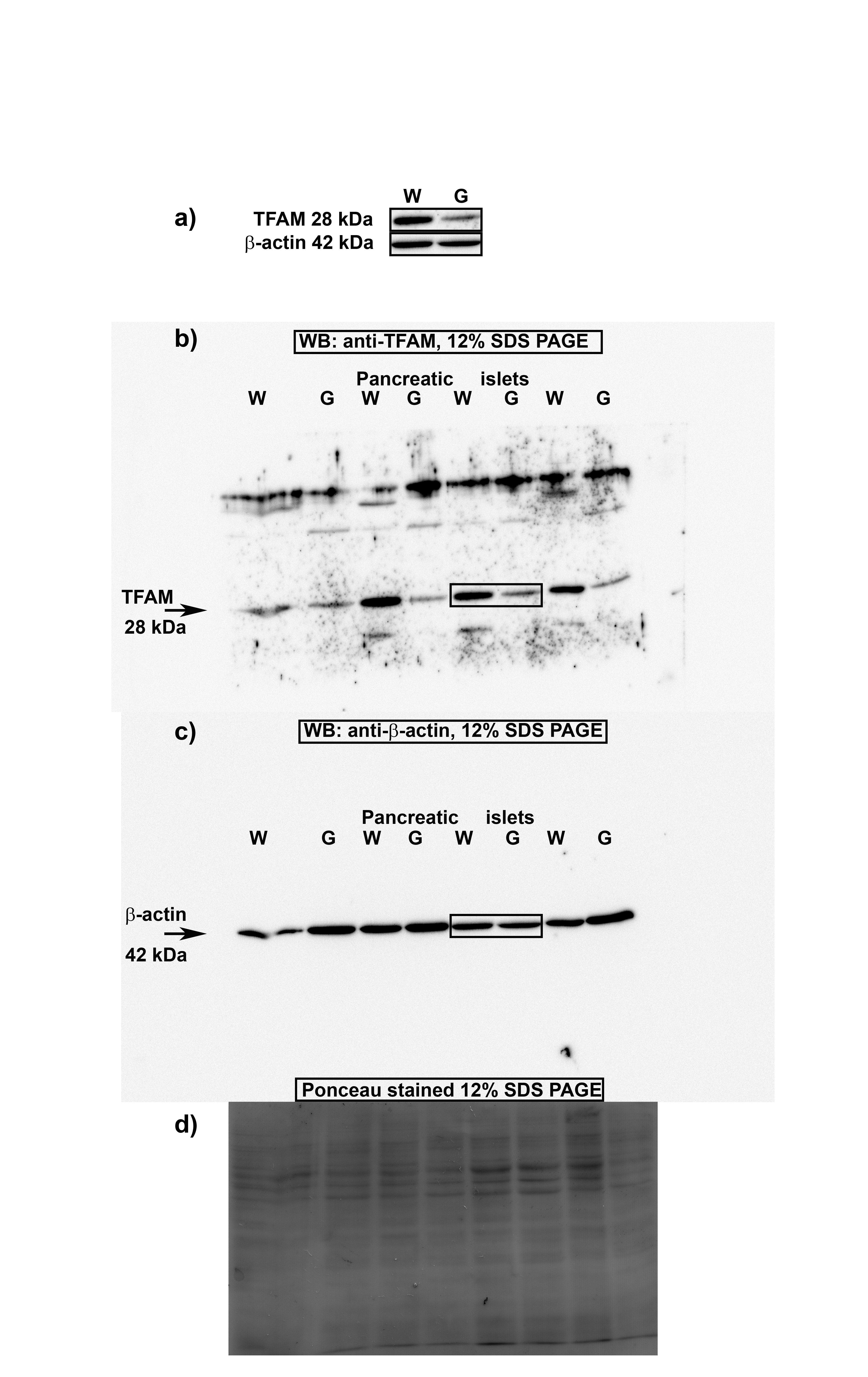
Representative Western blots: **a) final cropped regions** shown in Fig.1d); **b) Original whole membrane** of Western blot developed with anti-TFAM antibodies; **c) the same membrane** reprobed with anti-β-actin antibodies; **d) the same membrane** stained by Ponceau Red. Boxes areas indicated the cropped regions. “W” – Wistar rat, 55 weeks old; “G” – Goto Kakizaki rat 55 weeks old.

**Supplemental Figure S2 Expression of mtSSB** Representative Western blots: **a) final cropped regions** shown in Fig.1d); **b) Original whole membrane** of Western blot developed with anti-mtSSB antibodies; **c) same**
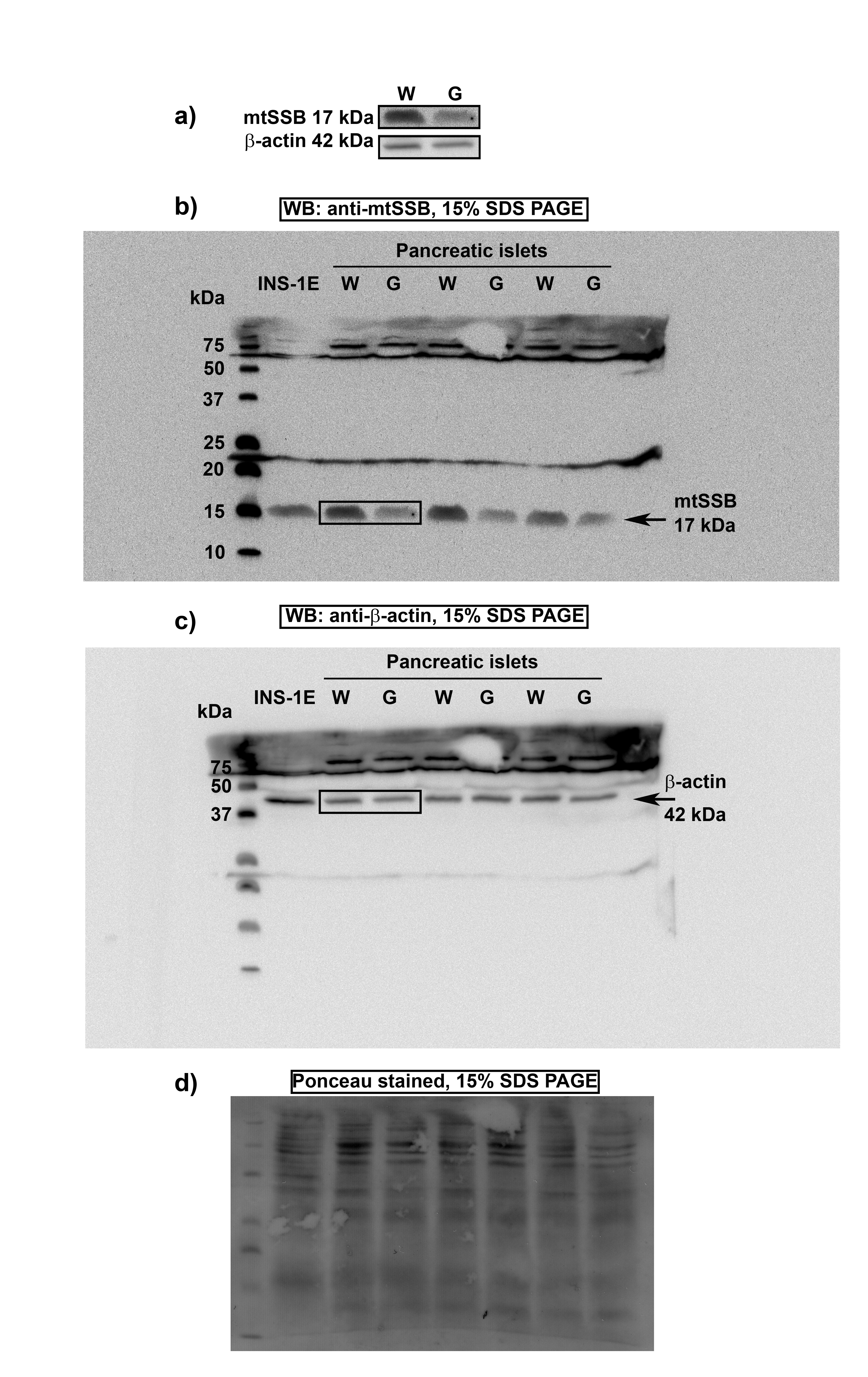
**membrane** reprobed with anti-β-actin antibodies; **d) same membrane** stained by Ponceau Red. Boxes areas indicated the cropped regions. “W” – Wistar rat, 55 weeks old; “G” – Goto Kakizaki rat 55 weeks old. INS-1E cells were used as an etalon.

**Suplemental Figure S3 Expression of PGC1α** Representative Western blots: **a) final cropped regions** shown in Fig.1d); **b) Original whole membrane** of Western blot developed with anti-PGC1α antibodies; **c) the same membrane** reprobed with anti-β-actin antibodies; **d) the same membrane** stained by Ponceau Red. Boxes areas indicated the cropped regions. “W” – Wistar rat, 55 weeks old; “G” – Goto Kakizaki rat 55 weeks old.

**
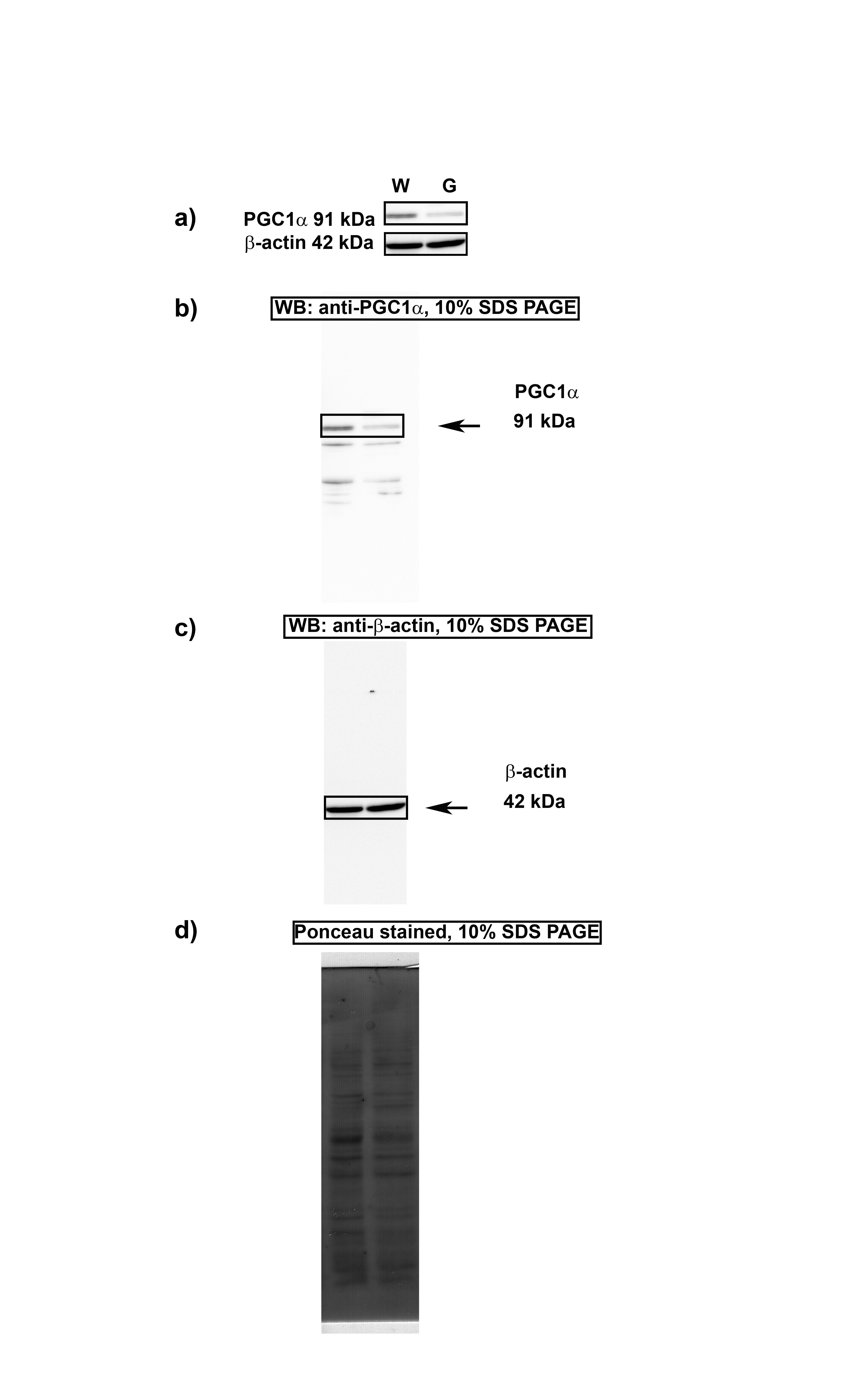
**

**Supplemental Figure S4 Expression of Nkx6.1** Representative Western blots: **a) final cropped regions** shown in Fig.7b); **b) Original whole membrane** of Western blot developed with anti-Nkx6.1 antibodies; **c) the same membrane** reprobed with anti-β-actin antibodies; **d) the same membrane** stained by Ponceau Red. Boxes areas
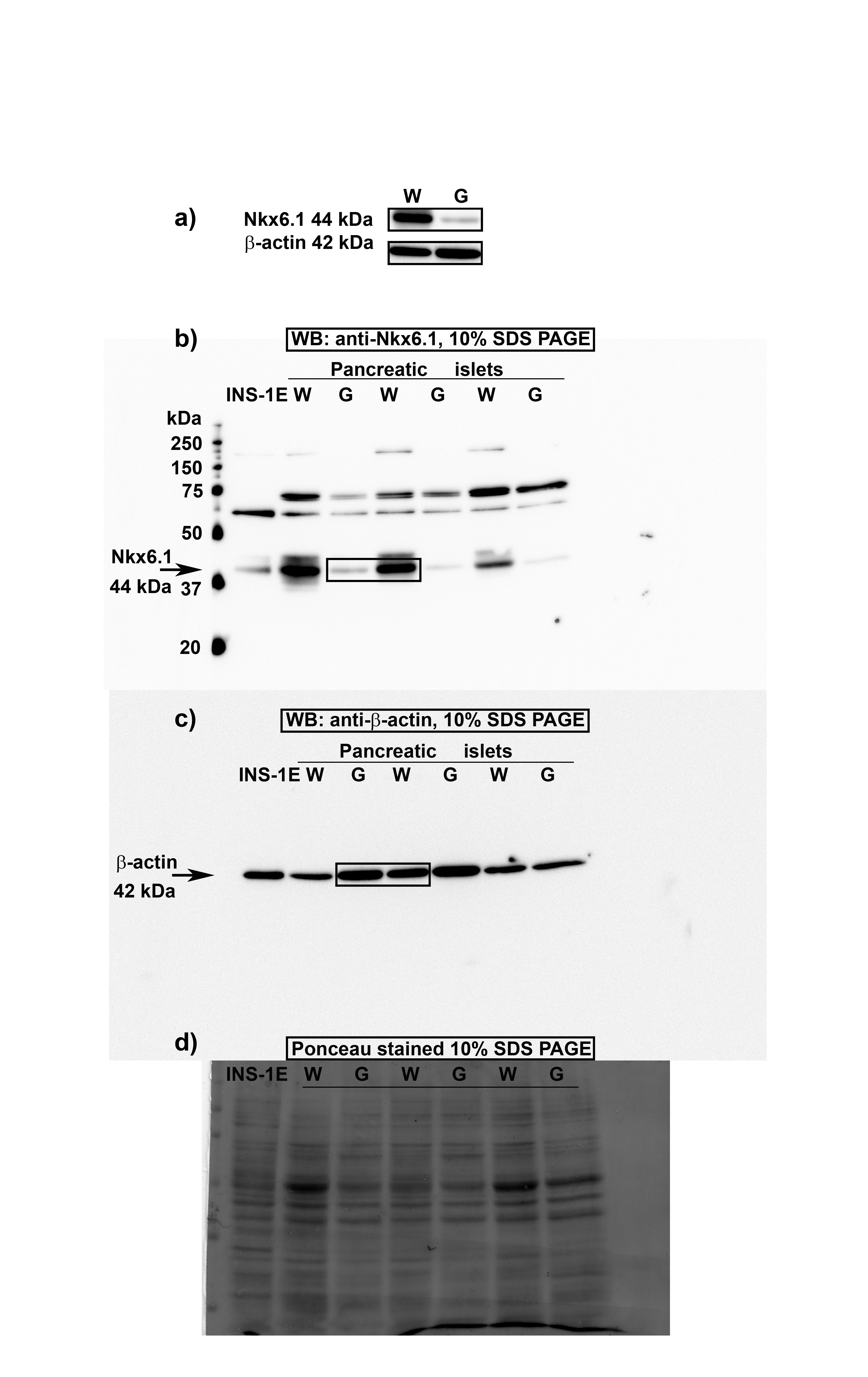
indicated the cropped regions. “W” – Wistar rat, 55 weeks old; “G” – Goto Kakizaki rat 55 weeks old. INS-1E cells were used as an etalon.
